# Brucine inhibits proliferation of glioblastoma cells by targeting the G-quadruplexes in the c-Myb promoter

**DOI:** 10.7150/jca.53689

**Published:** 2021-02-02

**Authors:** Qiaochu Liu, Qunhui Wang, Chuanqi Lv, Ziqiang Liu, Haijun Gao, Yong Chen, Gang Zhao

**Affiliations:** 1 Department of Neurosurgery, The First Hospital of Jilin University, Changchun, China.; 2 Clinical College, Jilin University, Changchun, China.

**Keywords:** brucine, c-Myb, G-quadruplex, glioblastoma

## Abstract

The proto-oncogene *c-Myb* plays an important role in cell proliferation, and its upregulation affects the development of glioblastomas. G-quadruplexes are secondary DNA or RNA structures that usually form in the promoter region of oncogenes, including *c-Myb*, and regulate the expression of these genes. The traditional Chinese medicine, brucine, is a ligand of the G-quadruplexes located in the promoter region of *c-Myb*. The present study investigated the therapeutic effects and mechanism of action of brucine in U87, LN18, and LN229 cells *in vitro* and *in vivo*. Our results showed that brucine suppressed the growth of these cells *in vitro* by arresting the cell cycle and reducing *c-Myb* expression. Dual-luciferase reporter assays showed that brucine inhibited *c-Myb* expression by targeting the guanine-rich sequence that forms G-quadruplexes in the *c-Myb* promoter. Moreover, U87 tumors were suppressed by brucine in a tumor xenograft nude mouse model. Therefore, brucine is potentially effective for treating glioblastomas.

## Introduction

Although cerebral tumors account for only ~2% of all cancers, their morbidity and mortality rates are high 1. Glioma is the most prevalent brain tumor and a major malignancy of the central nervous system in adults 2. Glioblastoma is one of the most aggressive types of gliomas with a World Health Organization grade of IV 3. Patients with glioblastoma are currently treated by tumor resection followed by radiotherapy and/or chemotherapy, typically with temozolomide 4. However, the 2-year survival rate achieved by this strategy is only 26.5%, and the median survival is 14.6 months 5. The prognosis of patients is poor because glioblastoma tumors are among the most resistant to radiation and cytotoxic chemotherapy 6. Thus, effective targets and practical treatment strategies for glioblastoma are needed.

The proto-oncogene *c-Myb* encodes C-MYB, which is an important transcription factor involved in cell proliferation and differentiation 7. This gene was discovered as a mammalian cellular homolog of *v-Myb* 8. High levels of *c-Myb* are expressed in many cancers, including colorectal tumors, T-cell leukemia, and most estrogen receptor-positive breast tumors 9. In addition, *c-Myb* is amplified and upregulated in several glioblastoma cell lines 10. Genes activated downstream of *c-Myb*, such as *Bcl-2*, indicate that *c-Myb* function is associated with cell survival 11. Furthermore, interactions between *c-Myb* and cyclin-D1 indicate that *c-Myb* is involved in cell cycle regulation 12. Therefore, *c-Myb* might serve as a therapeutic target for glioblastomas.

**Figure 1:**
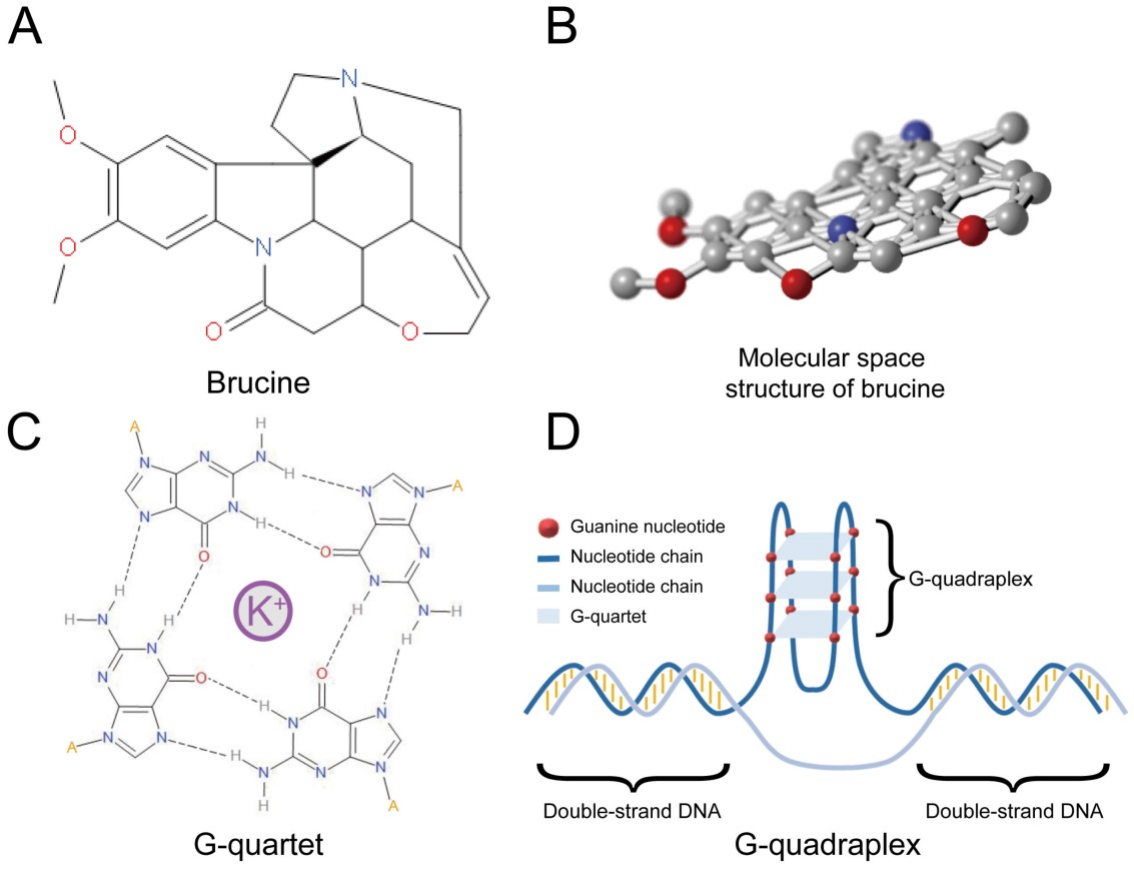
Structure of brucine was shown in Figure 1A and 1B. Molecular space structure of brucine is a conjugative flat. A G-quartet is formed by 4 guanine nucleotides surrounding a positive ion, usually K^+^ (C) and a G-quadruplex is formed by several G-quartets (D).

Polynucleotide secondary structures (G-quadruplexes) comprise several guanine tetrads called G-quartets (Figure 1C) that form layers through π-π stacking. G-quadruplexes (Figure 1D) can form rapidly under appropriate physiological conditions and interact with specific proteins that have either a stabilizing or unfolding function 13. Many G-quadruplexes are located in guanine-rich RNA or DNA sequences, and particularly in the promoter regions of oncogenes such as *c-Myc* 14. About 50% of human genes have G-quadruplexes near their promoters 15. However, G-quadruplexes are not evenly distributed, and are more prevalent in proto-oncogenes than in tumor suppressor genes 16. This notable bias suggests that G-quadruplexes are important for regulating the expression of genes, particularly proto-oncogenes. G-quadruplexes have the potential to serve as regulators of gene expression and therapeutic targets for specific cancers 17. For instance, G-quadruplexes of c-Myb negatively regulate its expression 18, suggesting that stabilization of these structures can suppress *c-Myb* transcription. Some small molecules that can interact with G-quadruplexes should stabilize these DNA structures 19. Brucine can form a conjugative flat in molecular space (Figure 1A and B), and it might be a stabilizing ligand of G-quadruplexes in the promoter region of *c-Myb* 20. Therefore, brucine might suppress *c-Myb* by binding to its G-quadruplexes in the promoter region. Here, we determined the anti-tumor effects and mechanism of action of brucine in the glioblastoma cell lines U87, LN18, and LN229.

## Materials and Methods

### Reagents and chemicals

Most reagents were purchased from MedChemExpress LLC. (Monmouth Junction, NJ, USA), and a few were obtained from other vendors as indicated. The following were purchased from specific vendors as follows: Brucine (APExBIO Technology LLC., Boston, MA, USA), antibodies (Abcam, Cambridge, UK) and the U87 human glioblastoma cell line (American Type Culture Collection, Manassas, VA, USA).

### Cell culture and harvest

Human U87, LN18, and LN229 glioblastoma cells were cultured in Gibco Dulbecco's Modified Eagle's Medium (DMEM, Thermo Fisher Scientific Inc., Waltham, MA, USA) supplemented with 10% fetal bovine serum and 100 U/mL of penicillin and streptomycin. The cells were maintained in a humidified incubator with 95% air atmosphere and 5% CO_2_ at a temperature of 37 °C. The cells were passaged for four generations, and then harvested as follows. The DMEM was removed, then the cells were washed with cold PBS, and detached from flasks using 1 mL of Trypsin (0.25%), Trypsin-EDTA (0.25%), or phenol red (Thermo Fisher Scientific Inc.). Digestion was terminated using DMEM, and then the cells were washed with cold PBS and centrifuged at 300 ×g for 5 min.

### MTT assays

The U87, LN18, and LN229 cells harvested in the log phase were adjusted to a density of 2.5 × 10^3^/mL, seeded (200 μL/well) into 96-well plates and incubated for 6 h. The medium in triplicate wells was replaced with 200 μL of DMEM containing 1, 0.5, 0.25, or 0 mM brucine, then the plates were incubated for 24 h. Thereafter 20 μL of MTT (5 mg/mL) was added to the wells, and plates were incubated for 4 h. The supernatant was removed, 150 μL of dimethyl sulfoxide was added, then the plates were shaken at 120 rpm for 10 min. The optical density (OD) 15 for each well was measured at λ 492 nm using an Epoch Microplate Spectrophotometer (BioTek Instrument, Inc., Winooski, VT, USA).

### Flow cytometry assays

U87, LN18, and LN229 cells were each incubated without (0 mM) or with 1-, 0.5-, or 0.25-mM brucine for 24 h, harvested, and counted. Annexin V/PI was determined using eBioscience^TM^ Annexin V-FITC Apoptosis Detection Kits (Thermo Fisher Scientific Inc.) as follows. The cells were washed with cold PBS. The supernatant was removed, then 1× binding buffer (100 μL) was added to each sample. Annexin V (5 μL) and PI (5 μL) were added, the volume of each sample was adjusted to 500 μL with 1× binding buffer. All these steps proceeded on ice. The cells were kept in the dark and analyzed using flow cytometry within 1 h. Cell cycles were assayed as follows. The cells were washed with cold (4 °C) PBS, centrifuged at 300 × g for 5 min, and then 70% alcohol was added. The cells were washed again with PBS, stained with propidium iodide 4 in the presence of 100 µL of RNase A for 30 min, and assessed using a CytoFLEX flow cytometer (Beckman Coulter, Inc., Brea, CA, USA).

### Quantitative real-time polymerase chain reaction (qPCR)

U87, LN18, and LN229 cells were each incubated without or with 1-, 0.5-, or 0.25-mM brucine for 24 h. Total cellular RNA was extracted using EasyPure® RNA Kits (TransGen Biotech, Beijing, China) according to the manufacture's protocol. The amount of total RNA was measured using an Epoch Microplate Spectrophotometer (BioTek Instrument, Inc.), then the RNA concentrations were adjusted to the same value using RNase-free water. Total RNA (1 µg/sample) was reverse-transcribed to cDNA using Superscript II Reverse Transcriptase (Thermo Fisher Scientific Inc.) as described by the manufacturer, SYBR Green, and an iCycler (Bio-Rad, Hercules, CA, USA). The reference was 100 μM Human GAPDH Endogenous Reference Gene Primers (Sangon Biotech, Shanghai, China). Primers targeting *c-Myb* (5′-GAAGCAGATTTTTCACCTAGCC-3′ and 5′-CTAGGTTCTCCTGCAGGTTTAG-3′) were designed in-house and synthesized at Sangon Biotech (Shanghai, China). Complementary DNA (2 μL) from each sample was added as the template in 18-μL reaction volumes containing 10 μL of Hieff qPCR SYBR Green Master Mix (YEASEN, Shanghai, China), 0.2 μM of the specific forward and reverse primers, and 7.2 μL of RNase-free water. qPCR proceeded using an Agilent Stratagene Mx3005P system (Agilent Technologies Inc., Santa Clara, CA, USA) according to the following protocol: 95 °C for 5 min, 40 cycles of 95 °C for 10 s, 60 °C for 20 s, and 72 °C for 20 s. Expression levels were determined using the comparative CT method. The concentration of cDNA was adjusted, and qPCR was repeated until the final *c-Myb* CT values were 20-30.

### Western blot assays

U87, LN18, and LN229 cells were each incubated without (0 mM) or with 1-, 0.5-, or 0.25-mM brucine for 24 h. The cells were harvested, washed with cold PBS, and homogenized on ice in RIPA lysis buffer with 1% 100× protease inhibitor cocktail. After centrifugation at 12,000 × g for 15 min, total protein levels were measured in supernatants using standard bicinchoninic acid assays. Samples with equal total protein concentrations were separated on 10% sodium dodecyl sulfide-polyacrylamide gels, then transferred to polyvinylidene fluoride membranes. Nonspecific binding was blocked using 5% fat-free milk in Tris-buffered saline with Tween 20 for 2 h at 20℃. The membranes were separately incubated with primary antibodies (all from Abcam, USA) against c-Myb (#ab117635), β-tubulin (#ab6046), BAX (#ab3191), cyclin-D1 (#ab226977), cyclin-B1 (#ab215436), and caspase-3 (#ab90437) overnight at 4℃. After incubation, the membranes were washed with Tris-buffered saline containing Tween 20 and incubated for 2 h at 20 °C with anti-mouse (#7076P2) or anti-rabbit (#7074P2) secondary antibodies (Cell Signaling Technology, Danvers, MA, USA) according to the specific primary antibody. Protein bands were detected using a MiniChemi 610 Chemiluminescent/Fluorescent Imaging and Analysis System (Beijing Sage Creation Science Co, Beijing, China).

### Dual-luciferase reporter assays

Transfected cells were assessed using a Dual-Luciferase Reporter Assay System (Promega Corp., Madison, WI, USA). The guanine-rich sequence, 5′-GGGCTGGGCTGGGCGGGG-3′, located in the c-Myb promoter region was synthesized to replace the sequence at the KpnⅠ site with NheⅠ of pGL3-basic (Promega Corp.), the vector of luciferase reporter gene 20. According to the protocol supplied by the manufacturer, pGL3-basic has a number of consensus transcription factor binding sites that express luciferase in complex environments. We seeded 293 cells in 12-well plates and co-transfected them with 1.0 μg of modified pGL3 (pGL3-p) or pGL3-basic and 0.2 μg of pRL-SV40 (Promega Corp.) for 48 h as described by the manufacturer. The cells were then incubated with or without (0 mM) or with 1-, 0.5-, or 0.25-mM brucine for 24 h.

### Electrospray ionization mass spectrometry (ESI-MS)

The sequence, 5′-GGGCTGGGCTGGGCGGGG-3′ (S1), and the complementary strand (S2), were synthesized (Sangon Biotech, Shanghai, China) and purified by high-performance liquid chromatography. Annealing buffer comprised Tris (10 mM), EDTA (1 mM), NaCl (50 mM), and pure water. Samples of DNA were divided into groups A and B with 0.1 mM S1, and groups C and D with 0.1 mM of both S1 and S2 in annealing buffer. Groups B and D were heated to, and maintained at 94 °C for 2 min, and gradually cooled to room temperature. Each group was further subdivided into groups that were incubated with various concentrations of brucine (A1, B1, C1, and D1, 0 mM; A2, B2, C2, and D2, 5 mM; A3, B3, C3, and D3, 10 mM; and A4, B4, C4, and D4, 20 mM). The results were determined using a SYNAPT G2-Si spectrophotometer (Waters, Milford, MA, USA).

### Tumor xenograft model in nude mice

Six-week-old female nude mice weighing 25-30 g (Animal Center of the Chinese Academy of Sciences, Shanghai, China) were allowed free access to food and water under controlled conditions (12/12 h light/dark cycle, 60% ± 5% humidity, 22 ± 3 °C). The Ethics Committee of the First Hospital of Jilin University and its institutional animal-care committee approved all experimental protocols. U87 cells (~ 5 × 10^6^) were suspended in 200 μL ECM-gel (Sigma-Aldrich, St. Louis, MO, USA) with 50% 1× PBS and injected subcutaneously into the right forelimb axilla of the mice. Two weeks later, mice with palpable tumors were randomly assigned to groups that were treated with either brucine or an equivalent volume of normal saline (NS). The mice in the experimental group were intraperitoneally injected with brucine (10 mg/kg) every 24 h for 10 days, during which, the mice were weighed, and tumors were measured every two days. All mice were sacrificed at the end of the experimental period and tumors were resected and stored at -80 °C. All animal manipulations proceeded according to the China Association for the Accreditation of Laboratory Animal Care guidelines for the humane treatment of animals and adhered to national and international standards.

### Data analyses

Data were statistically analyzed using GraphPad Prism 6 software version 6.02 (GraphPad, Inc., La Jolla, CA, USA) and are presented as means ± standard deviation. Two or more groups were compared using Student *t-*tests and one-way analysis of variance, respectively. Results with *P* < 0.05 were considered statistically significant.

## Results

### Brucine inhibited glioblastoma growth *in vitro*

**Figure 2:**
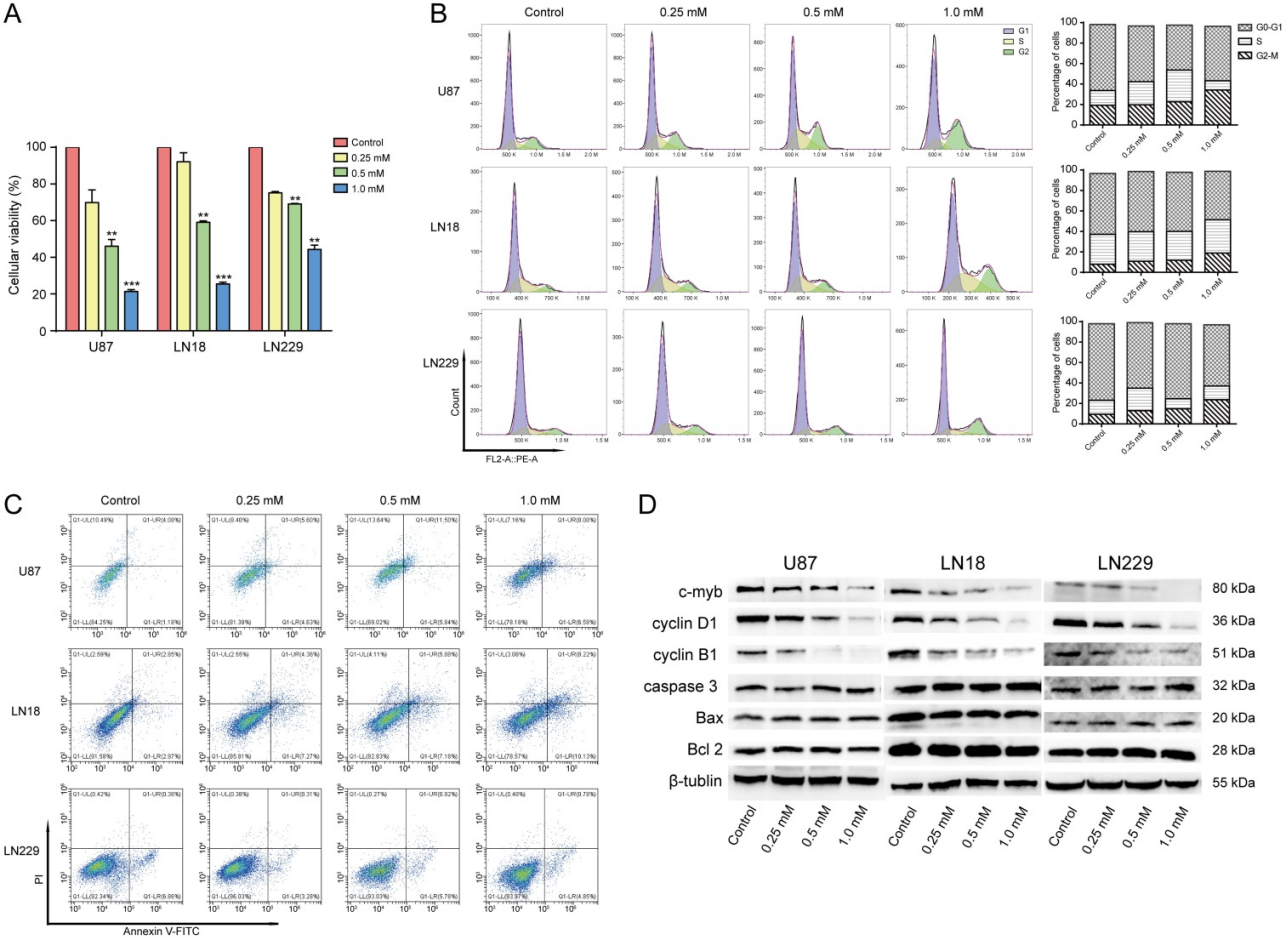
Brucine inhibited glioblastoma *in vitro*. U87, LN18, and LN229 cells were treated with brucine at different concentrations for 24 h then assessed using MTT assay (A) or flow cytometry (B and C) for cell cycle and apoptosis or used for protein extraction. The cell viability is represented by the OD 450 nm values shown in the Figure 2A. The overall results are presented as the mean ± SD and analyzed using a one-way ANOVA test and *P* < 0.0001. Each treatment group is separately compared to the control group and analyzed by the *t*-test. The results of cell cycle and apoptosis are shown in the B and C. The expression levels of c-Myb, Cyclin B1, Cyclin D1, BAX, Bcl-2 and Caspase-3 proteins extracted from the cells were detected using western blotting (D). β-Tubulin was used as an internal reference.

The effect of brucine on the viability of the three cell lines was assessed using MTT assays. Figure 2A shows that brucine negatively affected the overall survival of cells. Specifically, the number of live cells was dose-dependently reduced. Tumor cells were significantly suppressed by brucine concentrations of 0.5 or 1.0 mM, compared with the control (0 mM) group. The mechanism through which brucine inhibited the survival of glioblastoma cells was assessed using flow cytometry. Treated or untreated tumor cells were examined using FACS to detect apoptosis and the cell cycle stage. Figure 2B shows that the ratio (%) of cells in the G2/M stage gradually became upregulated with increasing amounts of brucine and was maximal at 1.0 mM, suggesting that brucine arrested the glioblastoma cell cycle, which inhibited glioblastoma cell growth. However, Figure 2C shows that the rate of apoptosis did not significantly correlate with the dose of brucine, indicating that brucine suppressed glioblastoma cell proliferation, rather than killing them.

The MTT and flow cytometry assays showed that 1.0 mM brucine decreased the proliferation of U87, LN18, and LN229 cells without apparently increasing cell death. We assessed the effects of brucine on cell cycle-related cyclin B1 and cyclin D1 and apoptosis-related BAX, Bcl-2, and caspase-3 protein levels using western blotting to understand this phenomenon. Levels of cyclin B1 and D1 gradually decreased with increasing doses of brucine, whereas BAX, Bcl-2, and caspase-3 levels were not significantly affected (Figure 2D). These results further validated our flow cytometry data.

### Brucine affects transcription and expression of *c-Myb*

**Figure 3:**
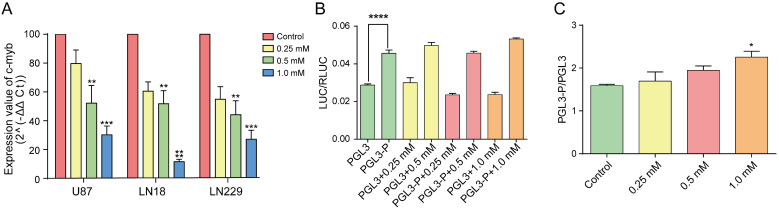
Brucine reduced c-Myb transcription. U87, LN18, and LN229 cells were treated with different concentrations of brucine and incubated for 24 h before their total RNA was extracted. GADPH was used as an internal reference. After reverse transcription of the total RNA, the cDNA was assessed using qPCR (A). The guanine-rich sequence in the *c-Myb* promoter region was synthesized to replace the sequence from site KpnⅠ to NheⅠ of pGL3-basic. 293 cells were transfected with pGL3-p or pGL3 and then treated with different concentrations of brucine for 24 h. pRL-SV40 was used as an internal reference. The results of LUC/RLUC (B) show the levels of the guanine-rich sequence and brucine affecting the plasmid expression, and the result of pGL3-p/pGL3 (C) represent how much the brucine influenced the guanine-rich sequence. The data are presented as the mean ± SD and analyzed using one-way ANOVA.

We investigated the effects of brucine on *c-Myb* transcription and expression using qPCR and western blotting. Total RNA was extracted from treated or untreated cells to detect *c-Myb* transcription using qPCR. The dose of brucine negatively correlated with and levels of *c-Myb* transcripts in U87, LN18, and LN229 cells (Figure 3A). Analysis of protein levels (Figure 2D) showed a similar downward trend in *c-Myb* expression in cells with increasing doses of brucine. These results suggested that brucine negatively regulates *c-Myb* expression via a direct or indirect pathway.

We examined whether brucine acts directly on the guanine-rich sequence located in the promoter region of *c-Myb* using dual-luciferase reporter assays. The promoter region of pGL3, 5′-GAGCTCTTACGCGT-3′, which did not contain a sufficient number of guanines to form a G-quadruplex, was replaced with the guanine-rich sequence at the *c-Myb* promoter region. Cells were transfected with the modified pGL3 (pGL3-p or pGL3-basic) vectors. Comparisons of the two untreated groups (Figure 3B) showed that pGL3-p expression was remarkably increased relative to pGL3-basic, indicating that the guanine-rich sequence with the potential to form G-quadruplexes significantly increased vector expression. The degree of influence of this sequence is represented by the ratio of pGL3-p/pGL3-basic under the same conditions. Figure 3C shows that the ratios increased with increasing concentrations of brucine, whereas pGL3-basic expression did not increase. This showed that the guanine-rich sequence in the promoter region of pGL3-basic was the only or at least the most effective target of brucine. Therefore, brucine might target the guanine-rich sequence of the *c-Myb* promoter and enhance its effect on pGL3 expression.

### Brucine binds G-quadruplexes to inhibit the formation of double-stranded DNA

**Figure 4:**
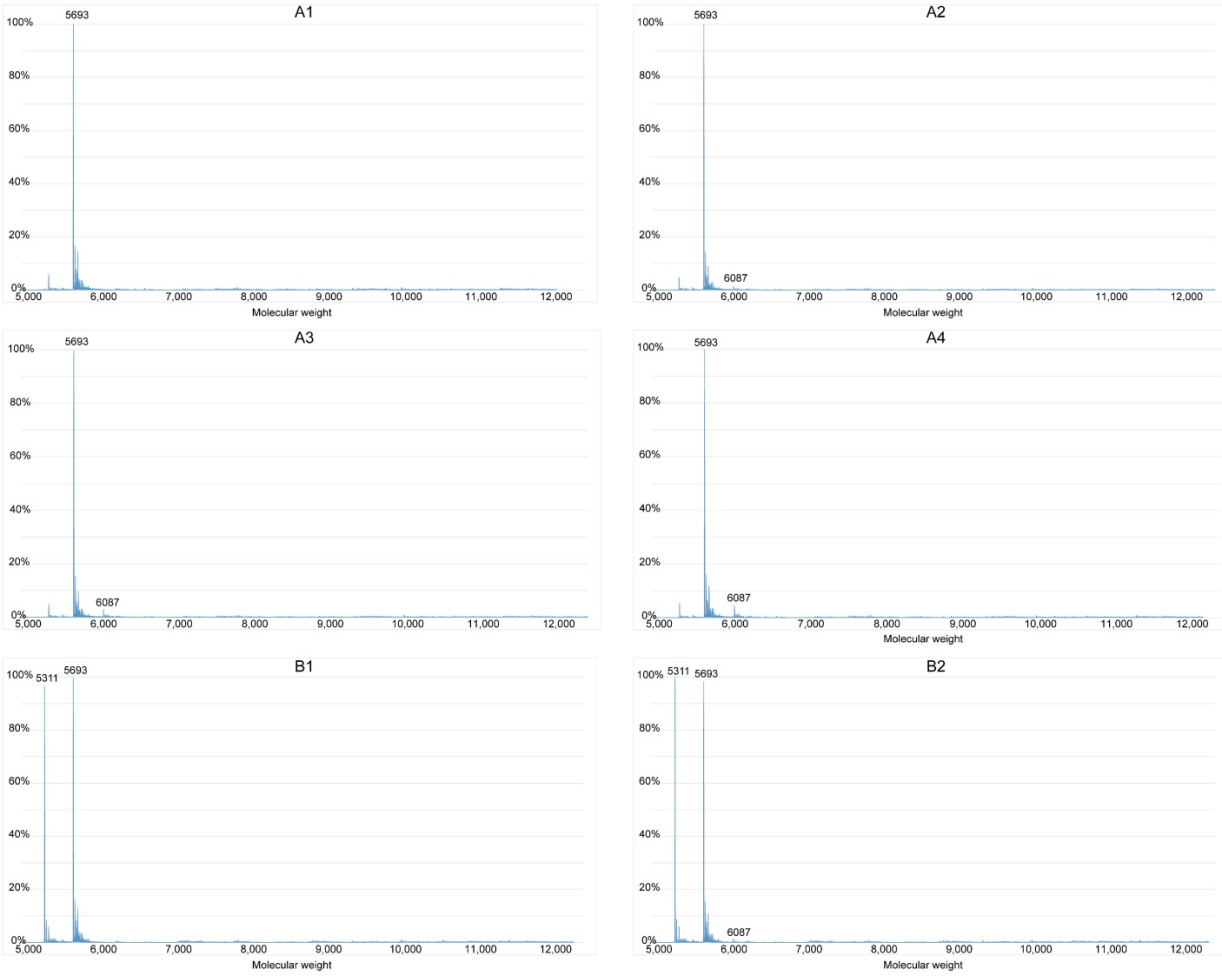

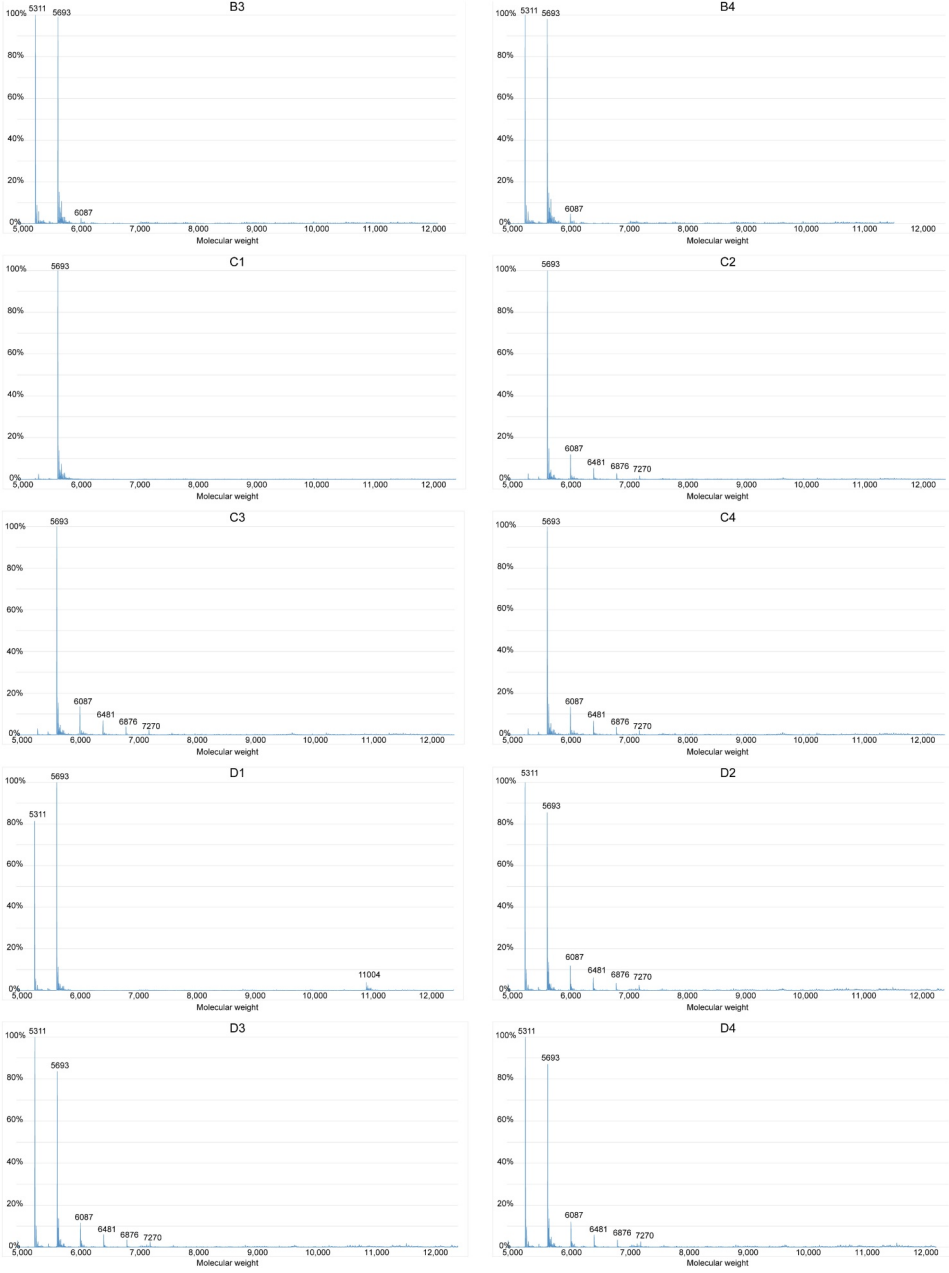
Brucine binds G-quadruplexes to inhibit the formation of double-stranded DNA. The guanine-rich sequence of the *c-Myb* promoter, S1, and its complementary strand, S2, were grouped and added to the annealing buffer. Samples were mixed with different concentrations of brucine and groups B and D were subject to the annealing process. Data were detected with a Waters SYNAPT G2-Si. The X-axis represents the molecular weights of the granules detected, and the Y-axis represents the ratios of the granules' quantities.

We investigated whether brucine binds G-quadruplexes formed by the guanine-rich sequence in the *c-Myb* promoter region using ESI-MS. The approximate molecular weights of S1, S2, double-stranded S1 and S2 (S1+S2), and brucine were 5693, 5311, 11004, and 394, respectively; therefore, the peak of a specific number in Figure 4 indicates the content of a particle with a molecular weight equal to the number. For instance, the peak at 6087 represents S1 with one molecule of bound brucine (S1+1b), and the peak at 6876 represents S1 with 3 molecules of bound brucine (S1+3b). The process of annealing might have helped S1 and S2 to form secondary structures such as G-quadruplexes and double-stranded structures. Without the annealing process, the results of groups A and C showed that only a very small amount of S1 combined with brucine in a ratio of 1:1 and double-stranded DNA was difficult to detect. Compared with the results of group A2-4, group B2-4 had peaks of S1+2b to S1+5b and higher peaks of S1+1b, suggesting that brucine was more likely to bind to a secondary structure of the guanine-rich S1, specifically G-quadruplexes of S1, rather than to the linear-like S1. D1 showed some formation of double-stranded S1+S2 without brucine rather than C1. However, S1+S2 peaks were essentially absent, so the peaks of S1+1b to S1+5b in D2-4 suggest that brucine disturbs the formation of S1+S2 by binding to the G-quadruplexes of S1.

### Brucine inhibits glioblastoma growth *in vivo*

**Figure 5:**
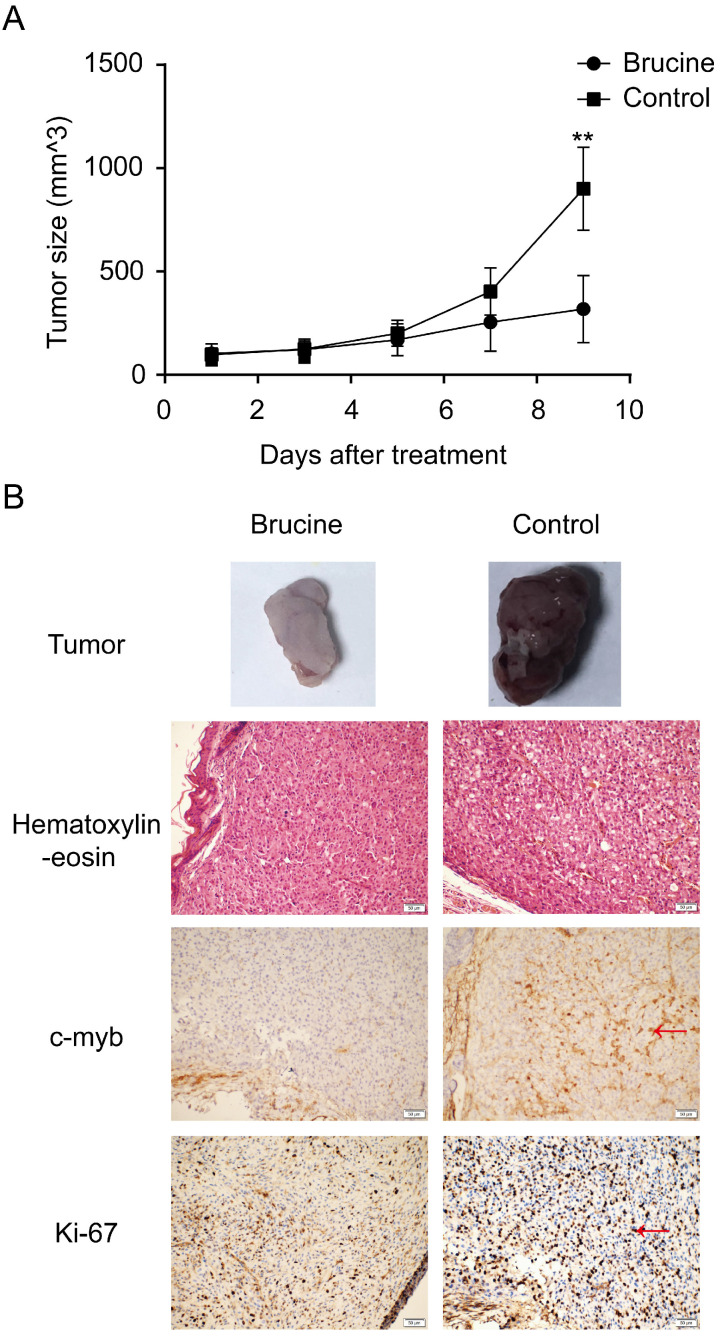
Brucine suppressed U87 tumors *in vivo*. U87 cells were injected into nude mice to establish an *in vivo* model. After tumor growth, the mice were separated into two groups and treated with brucine or NS. The changes in tumor sizes are shown (A) as the mean ± SD. The typical resected tumors are shown in (B) and Ki-67 and c-Myb expression levels in them were detected by immunohistochemistry and images were captured at 200× magnification (B).

To determine whether brucine has antitumor effects on glioblastoma both *in vitro* and *in vivo*, we established a U87 xenograft model using female nude mice. Tumors were palpable in all mice at 2 weeks after injecting equal amounts of U87 cells. The mice were separated into brucine and control groups. All mice remained alive without obvious side effects throughout the experimental period. Changes in tumor size in the two groups were calculated using the formula, *tumor volume* = *1/2* × *length* × *width*^2^ 21. Figure 5A shows that the average tumor size increased more slowly in the brucine group than in the control group. Furthermore, immunohistochemical assessment revealed lower levels of both *Ki-67* and *c-Myb* in the tumors from the group administered with brucine than those in the control group (Figure 5B). Therefore, brucine has therapeutic effects on U87 tumors in mouse xenograft models.

## Discussion

Glioblastomas comprise one of the most fatal and chemotherapy-resistant malignancies of the central nervous system. Here, brucine inhibited the proliferation of glioblastoma cells *in vitro* and *in vivo*. Other studies of brucine have indicated that the mechanisms underlying its anti-tumor effects include cell cycle arrest and induction of apoptosis in cancers such as hepatocellular carcinoma 22, multiple myeloma 23, and colon cancer 24. However, brucine (1.0 mM) did not significantly induce the apoptosis of glioblastoma cells *in vitro*, suggesting that brucine acts on different tumors through different pathways, or that glioblastomas are not as sensitive to brucine as other cancer cells, particularly in terms of susceptibility to apoptosis. Although increasing the concentration of brucine would probably lead to more apoptosis, we maintained a low brucine dose because of its considerable toxicity (intravenous toxicity of brucine in mice: LD_50_ = 13.17 mg/kg, LD_5_ = 9.17 mg/kg) 25. Moreover, we intraperitoneally administered a relatively safe dose (10 mg/kg) of brucine *in vivo*. None of the mice died due to brucine toxicity. The tumor sizes *in vivo* indicated the therapeutic effect of brucine on glioblastoma. In addition, immunohistochemistry of the tumors revealed reduced *c-Myb* protein levels in the brucine group. Combined with the results of qPCR and western blotting, *c-Myb* appears to play an important role in the mechanism underlying the effects of brucine on glioblastoma.

Parallel G-quadruplex structures in various genes form based on their unique DNA sequences, and they can interact with specific proteins or small molecules 13. The same sequence can form the same G-quadruplexes and interact with the same molecules in different genes. Our ESI-MS assays showed that brucine bound to the secondary structure of S1, G-quadruplexes, and blocked the formation of double-stranded DNA of S1 and S2. Typically, guanine-rich sequences do not easily form double-stranded DNA with its complementary strand because of G-quadruplexes format during the annealing process. Brucine enhanced the formation of G-quadruplexes to amplify this issue, affecting the transcription of specific genes. However, although brucine targeted the specific guanine-rich sequence from the c-Myb promoter, dual-luciferase assays showed that it had different its effects on G-quadruplexes in *c-Myb* and pGL3-p; brucine inhibited* c-Myb* and functioned as an agonist of pGL3-p. In addition, only the guanine-rich sequence in the pGL3-p promoter enhanced plasmid expression compared with pGL3-basic that was not treated with brucine. This might be due to different positions of G-quadruplexes in their DNA, different transcription factors associated with c-Myb and pGL3, and different proto-oncogene and pGL3 plasmid sequences. Further studies of the function of different G-quadruplexes in plasmids and non-oncogenes are needed to resolve these differences.

We also showed that the *c-Myb* oncogene in glioblastoma is inhibited by interactions between brucine and G-quadruplexes in the *c-Myb* promoter, which resulted in reduced cell proliferation *in vitro*. This suggested that other ligands targeting the same structure have similar glioblastoma cell anti-tumor effects. For example, dehydrocorydaline has greater binding affinity and selectivity for c-Myb G-quadruplexes than brucine 26. Thus, dehydrocorydaline is another potential anti-tumor drug for glioblastoma that might be more effective and selective, and less toxic than brucine. However, the therapeutic effects of dehydrocorydaline on glioblastomas has not been investigated. We propose that the therapeutic efficiency and safety should be compared between dehydrocorydaline and brucine for treating glioblastoma.

## Conclusions

Brucine is an effective suppressor of glioblastoma *in vitro* and *in vivo*. The glioblastoma cell cycle was arrested and c*-Myb* oncogene expression was reduced by targeting G-quadruplexes located in the *c-Myb* promoter region. This is an important therapeutic mechanism indicating that brucine could be an effective therapy for glioblastoma.

## Abbreviations

ESI-MS: electrospray ionization mass spectrometry
OD: optical density
NS: normal saline
PI: propidium iodide

## References

[1] Jacobs JF, Idema AJ, Bol KF, Nierkens S, Grauer OM, Wesseling P (2009). Regulatory T cells and the PD-L1/PD-1 pathway mediate immune suppression in malignant human brain tumors. Neuro-oncology.

[2] Ostrom QT, Bauchet L, Davis FG, Deltour I, Fisher JL, Langer CE (2014). The epidemiology of glioma in adults: a “state of the science” review. Neuro-oncology.

[3] Louis DN, Perry A, Reifenberger G, von Deimling A, Figarella-Branger D, Cavenee WK (2016). The 2016 World Health Organization Classification of Tumors of the Central Nervous System: a summary. Acta neuropathologica.

[4] Weller M, van den Bent M, Tonn JC, Stupp R, Preusser M, Cohen-Jonathan-Moyal E (2017). European Association for Neuro-Oncology (EANO) guideline on the diagnosis and treatment of adult astrocytic and oligodendroglial gliomas. The Lancet Oncology.

[5] Stupp R, Mason WP, van den Bent MJ, Weller M, Fisher B, Taphoorn MJ (2005). Radiotherapy plus concomitant and adjuvant temozolomide for glioblastoma. The New England journal of medicine.

[6] Masui K, Cloughesy TF, Mischel PS (2012). Review: molecular pathology in adult high-grade gliomas: from molecular diagnostics to target therapies. Neuropathology and applied neurobiology.

[7] Oh IH, Reddy EP (1999). The myb gene family in cell growth, differentiation and apoptosis. Oncogene.

[8] Beug H, von Kirchbach A, Döderlein G, Conscience JF, Graf T (1979). Chicken hematopoietic cells transformed by seven strains of defective avian leukemia viruses display three distinct phenotypes of differentiation. Cell.

[9] Bell D, Roberts D, Karpowicz M, Hanna EY, Weber RS, El-Naggar AK (2011). Clinical significance of Myb protein and downstream target genes in salivary adenoid cystic carcinoma. Cancer biology & therapy.

[10] Welter C, Henn W, Theisinger B, Fischer H, Zang KD, Blin N (1990). The cellular myb oncogene is amplified, rearranged and activated in human glioblastoma cell lines. Cancer letters.

[11] Ramsay RG, Gonda TJ (2008). MYB function in normal and cancer cells. Nature reviews Cancer.

[12] Lei W, Liu F, Ness SA (2005). Positive and negative regulation of c-Myb by cyclin D1, cyclin-dependent kinases, and p27 Kip1. Blood.

[13] Yang D (2019). G-Quadruplex DNA and RNA. Methods in molecular biology (Clifton, NJ).

[14] Grand CL, Powell TJ, Nagle RB, Bearss DJ, Tye D, Gleason-Guzman M (2005). Mutations in the G-quadruplex silencer element and their relationship to c-MYC overexpression, NM23 repression, and therapeutic rescue. Proceedings of the National Academy of Sciences of the United States of America.

[15] Rhodes D, Lipps HJ (2015). G-quadruplexes and their regulatory roles in biology. Nucleic acids research.

[16] Eddy J, Maizels N (2006). Gene function correlates with potential for G4 DNA formation in the human genome. Nucleic acids research.

[17] Haider S, Parkinson GN, Marsh TC (2018). G-Quadruplexes (GQU). Journal of nucleic acids.

[18] Palumbo SL, Memmott RM, Uribe DJ, Krotova-Khan Y, Hurley LH, Ebbinghaus SW (2008). A novel G-quadruplex-forming GGA repeat region in the c-myb promoter is a critical regulator of promoter activity. Nucleic acids research.

[19] Francisco AP, Paulo A (2017). Oncogene Expression Modulation in Cancer Cell Lines by DNA G-Quadruplex-Interactive Small Molecules. Current medicinal chemistry.

[20] Li H, Hai J, Zhou J, Yuan G (2016). Exploration of binding affinity and selectivity of brucine with G-quadruplex in the c-myb proto-oncogene by electrospray ionization mass spectrometry. Rapid communications in mass spectrometry: RCM.

[21] Naito S, von Eschenbach AC, Giavazzi R, Fidler IJ (1986). Growth and metastasis of tumor cells isolated from a human renal cell carcinoma implanted into different organs of nude mice. Cancer research.

[22] Saraswati S, Alhaider AA, Agrawal SS (2013). Anticarcinogenic effect of brucine in diethylnitrosamine initiated and phenobarbital-promoted hepatocarcinogenesis in rats. Chemico-biological interactions.

[23] Rao PS, Ramanadham M, Prasad MN (2009). Anti-proliferative and cytotoxic effects of Strychnos nux-vomica root extract on human multiple myeloma cell line - RPMI 8226. Food and chemical toxicology: an international journal published for the British Industrial Biological Research Association.

[24] Zheng L, Wang X, Luo W, Zhan Y, Zhang Y (2013). Brucine, an effective natural compound derived from nux-vomica, induces G1 phase arrest and apoptosis in LoVo cells. Food and chemical toxicology: an international journal published for the British Industrial Biological Research Association.

[25] Chen J, Yan GJ, Hu RR, Gu QW, Chen ML, Gu W (2012). Improved pharmacokinetics and reduced toxicity of brucine after encapsulation into stealth liposomes: role of phosphatidylcholine. International journal of nanomedicine.

[26] Cui X, Yuan G (2011). Formation and recognition of G-quadruplex in promoter of c-myb oncogene by electrospray ionization mass spectrometry. Journal of mass spectrometry: JMS.

